# Comparative proteomic analysis provides insight into a complex regulatory network of taproot formation in radish (*Raphanus sativus* L.)

**DOI:** 10.1038/s41438-018-0057-7

**Published:** 2018-10-01

**Authors:** Yang Xie, Liang Xu, Yan Wang, Lianxue Fan, Yinglong Chen, Mingjia Tang, Xiaobo Luo, Liwang Liu

**Affiliations:** 1 National Key Laboratory of Crop Genetics and Germplasm Enhancement, Key Laboratory of Horticultural Crop Biology and Genetic Improvement (East China) of MOA, College of Horticulture, Nanjing Agricultural University, Nanjing, 210095 PR China; 2 The UWA Institute of Agriculture, UWA School of Agriculture and Environment, The University of Western Australia, Perth, WA 6001 Australia

## Abstract

The fleshy taproot of radish is an important storage organ determining its yield and quality. Taproot thickening is a complex developmental process in radish. However, the molecular mechanisms governing this process remain unclear at the proteome level. In this study, a comparative proteomic analysis was performed to analyze the proteome changes at three developmental stages of taproot thickening using iTRAQ approach. In total, 1862 differentially expressed proteins (DEPs) were identified from 6342 high-confidence proteins, among which 256 up-regulated proteins displayed overlapped accumulation in S1 (pre-cortex splitting stage) vs. S2 (cortex splitting stage) and S1 vs. S3 (expanding stage) pairs, whereas 122 up-regulated proteins displayed overlapped accumulation in S1 vs. S3 and S2 vs. S3 pairs. Gene Ontology (GO) and pathway enrichment analysis showed that these DEPs were mainly involved in several processes such as “starch and sucrose metabolism”, “plant hormone signal transduction”, and “biosynthesis of secondary metabolites”. A high concordance existed between iTRAQ and RT-qPCR at the mRNA expression levels. Furthermore, association analysis showed that 187, 181, and 96 DEPs were matched with their corresponding differentially expressed genes (DEGs) in S1 vs. S2, S1 vs. S3, and S2 vs. S3 comparison, respectively. Notably, several functional proteins including cell division cycle 5-like protein (CDC5), expansin B1 (EXPB1), and xyloglucan endotransglucosylase/hydrolase protein 24 (XTH24) were responsible for cell division and expansion during radish taproot thickening process. These results could facilitate a better understanding of the molecular mechanism underlying taproot thickening, and provide valuable information for the identification of critical genes/proteins responsible for taproot thickening in root vegetable crops.

## Root vegetables: tapping into root thickening for improved yields

An investigation into the proteins and genes responsible for taproot thickening in radish could help improve yields of root vegetables. The fleshy taproot of vegetables like radish are vital to food supplies worldwide, but the precise mechanisms behind healthy, high quality taproot formation, and thickening are unclear. Liwang Liu at Nanjing Agricultural University in Nanjing, China, and co-workers have identified several functional proteins and corresponding genes responsible for cell division in taproot growth and thickening. The team analyzed changes in protein expression at three different developmental stages of radish taproot. They identified 1862 differentially expressed proteins involved in several processes including sugar and starch metabolism, hormone signaling, and biosynthesis of metabolites. Their results provide fundamental insights into the regulation of taproot thickening in radish and could be expanded to other root vegetables.

## Introduction

Radish (*Raphanus sativus* L., 2*n* = 2× = 18), one of the most important worldwide root vegetable crops, is an annual or biennial herb belonging to Brassicaceae family. The fleshy taproot is the significant edible part in radish plants, which contains abundant nutrient substances such as carbohydrates, crude fiber, vitamin C, protein, and other secondary metabolites including glucosinolate, and it determines the final yield and quality^1^. In addition, it is of significant value on diet and medicine^2–5^. Therefore, it is urgent to clarify the molecular mechanism underlying taproot thickening in radish plants.

In the past years, the morphological, physiological, and anatomical characterization of taproot thickening have been extensively studied on radish^6,7^. The morphogenetic process displays dynamic changes in the period of taproot thickening, which is determined by the interactions of genetic, environmental, and physiological factors^8,9^. Essentially, fleshy taproot development is the result of related genes programmed expression^8,9^. In recent years, with the rapidly developed “omics” technology, the draft genome sequences and transcriptome studies of *R. sativus* have been reported, which provide a valuable database for identification of the critical genes and genetic manipulation in radish^10–13^. Using RNA-Seq technique, characterization of transcriptome and miRNA may dissect the molecular mechanism underlying taproot thickening, with several miRNAs and differentially expressed genes (DEGs) identified during three different stages of taproot thickening (pre-cortex splitting stage, cortex splitting stage, and expanding stage)^8,14^. However, the molecular mechanism underlying taproot thickening in radish has not been comprehensively uncovered at the proteome level.

Proteomics studies provide a powerful tool for exploring related proteins in specific tissues with different stages in the post-genomic era, therefore comparative proteomic analysis for identifying proteins involved in radish taproot thickening would be an indispensable way for complementing genomics analysis to further explore molecular mechanism governing radish taproot thickening. Currently, the two-dimensional gel electrophoresis (2-DE) in combination with mass spectrometry (MS) has been employed for proteomic studies providing useful tools for protein separation and quantification^15–18^. However, it has some limitations in protein abundance identification. More recently, isobaric tags for relative and absolute quantitation (iTRAQ), a new and powerful technology, was used in quantitative proteomics by isotope tagging and high-performance liquid chromatography (HPLC)^19,20^. High sensitivity (less than 1 ppm), low detection limit (proteins of less than 10 kD or greater than 200 kD) in identification and quantitation of proteins aspects make iTRAQ a valuable approach for proteomics studies in some plant species including cassava^21^, potato^22,23^, and *Brassica napus*^24^.

Previous studies reported that cortex splitting stage is a sign of transition from primary growth to secondary growth, and it is of significance to investigate the expression changes of proteins during the pre-cortex splitting stage, cortex splitting stage, and expanding stage of taproot thickening in radish^9,11^. In this study, comparative proteomic analysis of monitoring differentially expressed proteins (DEPs) from three libraries of pre-cortex splitting stage (S1, 10 days after sowing (DAS)), cortex splitting stage (S2, 20 DAS), and expanding stage (S3, 40 DAS) were conducted by iTRAQ-coupled LC-MS/MS. Furthermore, association analysis between DEGs and DEPs in three development stages of taproot thickening was performed for further screening of critical genes involved in taproot formation in radish. The outcomes of this study could be beneficial for further dissection of the molecular mechanism governing radish taproot thickening, and provide a fundamental basis for genetic improvement of taproot formation in root vegetable crops.

## Materials and methods

### Plant materials

Seeds of an advanced inbred line of radish (*R. sativus* L.) “NAU-YH” were germinated on moist filter paper in dark at 25 °C for 3 days, and then cultured in plastic pots with 16 h light (25 °C) and 8 h dark (18 °C) for the rest of the experimental period. At pre-cortex splitting stage (S1, 10 DAS), cortex splitting stage (S2, 20 DAS), and expanding stage (S3, 40 DAS), taproots were sampled, respectively, and three biological replicates were collected for each stage. Equal amounts of taproot samples from three independent biological replicates of each stage were pooled and immediately frozen in liquid nitrogen, then stored at −80 °C for proteomic analysis.

### Protein preparation

The taproot samples were ground in liquid nitrogen to a fine powder and extracted in lysis buffer, then 1 mM phenylmethyl sulfonyl fluoride (PMSF), 2 mM ethylene diamine tetraacetic acid (EDTA), and 10 mM dithiothreitol (DTT) were added. The samples were sonicated for 15 min and centrifuged at 25,000×*g* for 20 min. The supernatant was mixed well with 5× volume of chilled acetone and incubated at −20 °C for 2 h. After centrifugation at 16,000×*g* for 20 min, the supernatant was discarded and then repeated previous processes once. The protein concentrations were measured with the Bradford assay^25^, and 12% sodium dodecyl sulfate polyacrylamide gel electrophoresis (SDS-PAGE) was used for separating proteins and checking the proteins.

### iTRAQ labeling and SCX fractionation

The extracted total protein from each sample solution (100 μg) was digested with Trypsin Gold with a ratio of protein:trypsin = 20:1 (w/w) at 37 °C for 4 h, and then Trypsin Gold (protein:trypsin = 20:1) added once more at 37 °C for 8 h. After trypsin digestion, peptides were dried by vacuum centrifugation and reconstituted in 0.5 M tetraethyl-ammonium bromide (TEAB) with the next steps following the manufacturer’s procedure of 8-plex iTRAQ reagent (Applied Biosystems). Briefly, 1 U of iTRAQ reagent was thawed and reconstituted in 70 μL isopropanol. Samples from pre-cortex splitting stage S1, cortex splitting stage S2, and expanding stage S3 were labeled with isobaric tags 116, 117, and 118, respectively, and incubated at room temperature for 2 h, and then pooled and dried by vacuum centrifugation^26–28^.

Then the labeled peptides were separated by SCX chromatography using separation column of 4.6 × 250 mm (Ultremex SCX column) with LC-20AB liquid system. In detail, the mixed labeled peptides were reconstituted in 4 mL buffer A (25 mM NaH_2_PO_4_ in 25% ACN, pH 2.7), and gradient-eluted in buffer B at a flow rate of 1 mL/min in column using 5% buffer B (25 mM NaH_2_PO_4_, 1 M KCl in 25% ACN, pH 2.7), 7 min; 5–60% in 20 min; 60–100% and maintenance for 1 min; 5% maintenance for 10 min^29^. The eluted process was supervised under 214 nm absorption photometry. The columns were cleaned to eliminate salts using StrataX. The extracted liquid was lyophilized and stored at −80 °C.

### LC-ESI-MS/MS analysis

The peptide was dissolved in buffer C (5% ACN, 0.1% FA) and centrifuged at 20,000×*g* for 10 min. The supernatant (approximately 10 μL) was loaded on a trap column and then the peptides were eluted onto an analytical column by LC-20AD nanoHPLC (Shimadzu, Kyoto, Japan) according to the manufacturer’s instruction. In detail, the peptides were loaded at 8 μL/min for 4 min, then the gradient program was performed as follows: it was started from 2 to 35% in buffer D (95% ACN, 0.1% FA) at 300 μL/min, followed by a 5 min linear gradient to 60%, then a 2 min linear gradient to 80% and maintenance for 4 min, and finally return to 5% in 1 min^29^. Data were acquired by a TripleTOF 5600 System. The MS was operated with more than 30,000 resolutions. A sweeping collision energy was set for 35 ± 5 eV with a dynamic exclusion setting of 1/2 of peak width (15 s), ensuring that the same precursor ion was fragmented not more than twice.

### Protein identification

For peptide data analysis, raw data was employed with the Mascot search engine (Matrix Science, London, UK; version 2.3.02) against the *Raphanus_sativus* (224,406 sequences) protein sequence database (ftp://ftp.kazusa.or.jp/pub/radish/). For protein identification, a series of standard parameters were set as follows: 0.1 Da fragmented mass tolerance, 0.05 Da peptide mass tolerances and one max missed cleavage. To reduce the probability of false peptide identification, only peptides at the 95% probability were used for protein identification by a Mascot probability analysis, and each confident protein contains at least one unique peptide^28^. For protein quantitation, protein has at least two unique spectra, and the parameter “median” was considered as the standard of quantitative protein ratios^26,30^. The identification results of significant DEPs were filtered with *P*-value < 0.05 and fold change (FC) > 1.2^31^.

### Bioinformatics analysis

A hierarchical cluster analysis of the DEPs was carried out using Cluster 3.0 software. The similarity of proteins was calculated with Euclidean distance, and the average-linkage method was selected for clustering^32^. Functional annotations of the DEPs were employed by KOBAS 2.0 program search against the non-redundant (nr) protein database deposited in NCBI. The database of Kyoto Encyclopedia of Genes and Genomes (KEGG), Gene Ontology (GO), and Cluster of Orthologous Groups (COG) were performed to categorize and group the DEPs^31,33^.

### RT-qPCR analysis

Quantitative real-time PCR (RT-qPCR) was employed to validate the quality of iTRAQ results. Total RNAs from taproot samples including S1, S2, and S3 stages was extracted and reverse transcribed to cDNA following the manufacturer’s instructions (Tiangen Biotech Co., Ltd., China). Each reaction was carried out using 10 μL 2× SYBR green reaction mix, 2.0 μL diluted cDNA, and 0.2 μM of each primer in a total volume of 20 μL system. RT-qPCR amplification reactions were conducted on a Real-Time PCR Detection System (Bio-Rad iQ5, USA) following the reported protocol^34^. The specific primers used for RT-qPCR were designed with Beacon Designer 7.0 software (Premier Bio-soft International, USA), which are listed in Supplementary Table S1. Three replicates for each gene assay were performed and mRNA expression levels were normalized by *Rs-Actin* gene. The relative gene expression value was calculated with the 2^−∆∆*C*T^ method.

## Results

### Protein identification

Based on LC-ESI-MS/MS analysis, a total of 411,811 spectra were generated in this study, which included 95,124 matched spectra and 46,912 unique spectra. Totally 27,080 peptides containing 16,722 unique peptides as well as 6342 proteins were identified. Moreover, protein mass was dominantly enriched in 20–60 kDa, and the peptide number and peptide length were mainly distributed at the range of 1–10 and 6–29 (in amino acids), respectively (Supplemental Figure S1).

### Functional annotation of proteins

**Fig. 1:**
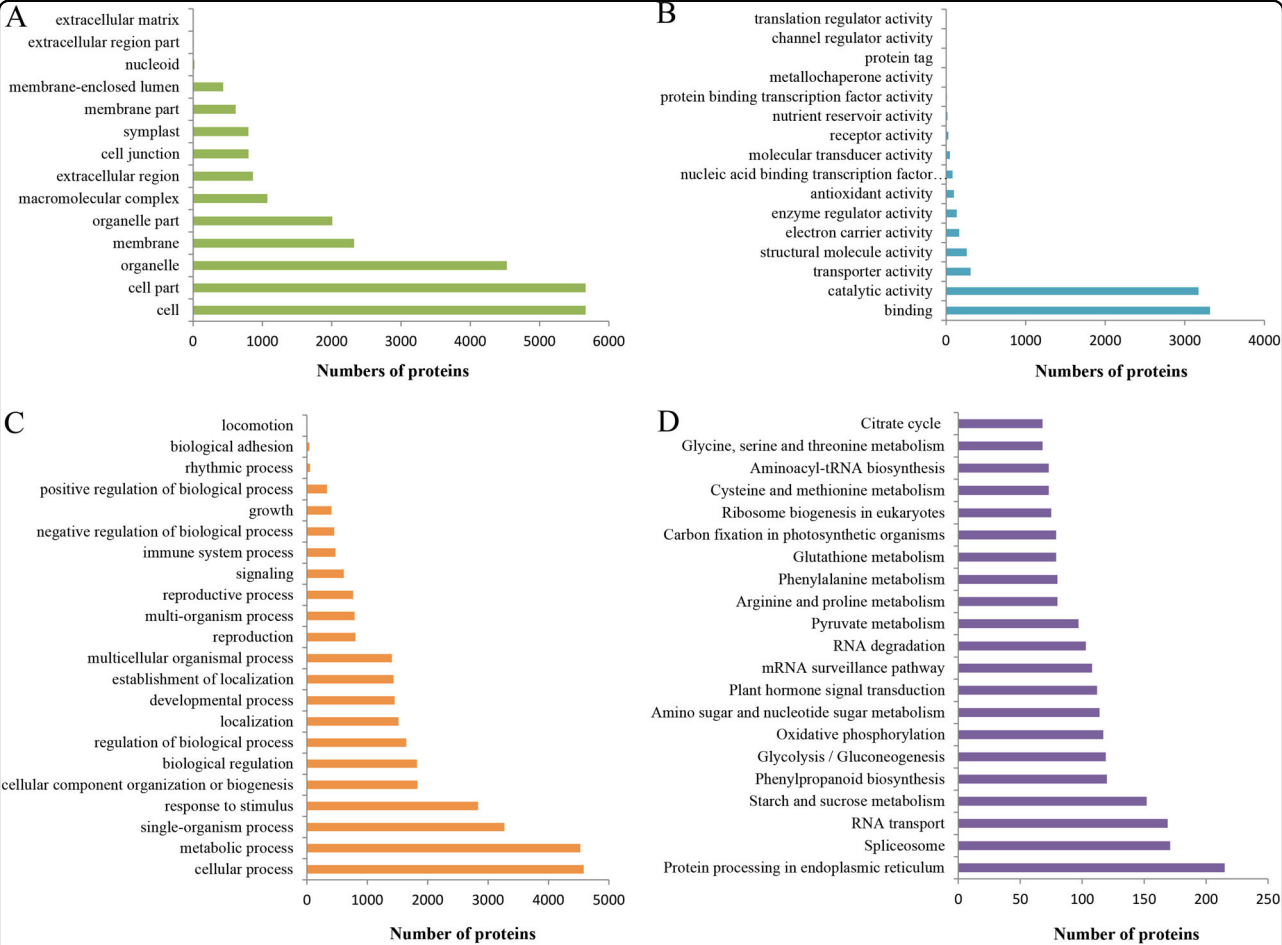
GO and KEGG pathway functional classification of the identified proteins. **a** The cell component category of GO classification. **b** The molecular function category of GO classification. **c** The biological function category of GO classification. **d** KEGG pathway functional classification and annotation

GO function classification showed that the proteins were mainly annotated to the terms of cellular process (14.77%, in biological process ontology), cell part (22.82%, in cell component ontology), and binding (43.40%, in molecular function ontology) (Fig. 1a–c).

KEGG pathway analysis revealed two dominant pathways: “Metabolic pathways” (ko01100) and “Biosynthesis of secondary metabolites” (ko01110). In addition, “Starch and sucrose metabolism” (ko00500) pathway was the seventh top of all, which was one of the most important pathways involved in taproot thickening (Fig. 1d)^13^.

The COG database was employed for protein orthologous classification, and a total of 5260 proteins were aligned to 24 COG terms. These results showed that the category of “general functions prediction only” (17.60%) was the largest, followed by “Posttranslational modification, protein turnover, chaperones” (12.36%), “Translation, ribosomal structure and biogenesis” (9.13%), “Carbohydrate transport and metabolism” (7.89%), and “Energy production and conversion” (7.13%). In contrast, the categories of “Cell motility” and “Nuclear structure” were less than five proteins with high homology (Supplemental Figure S2).

### Screening of differentially expressed proteins (DEPs)

**Fig. 2:**
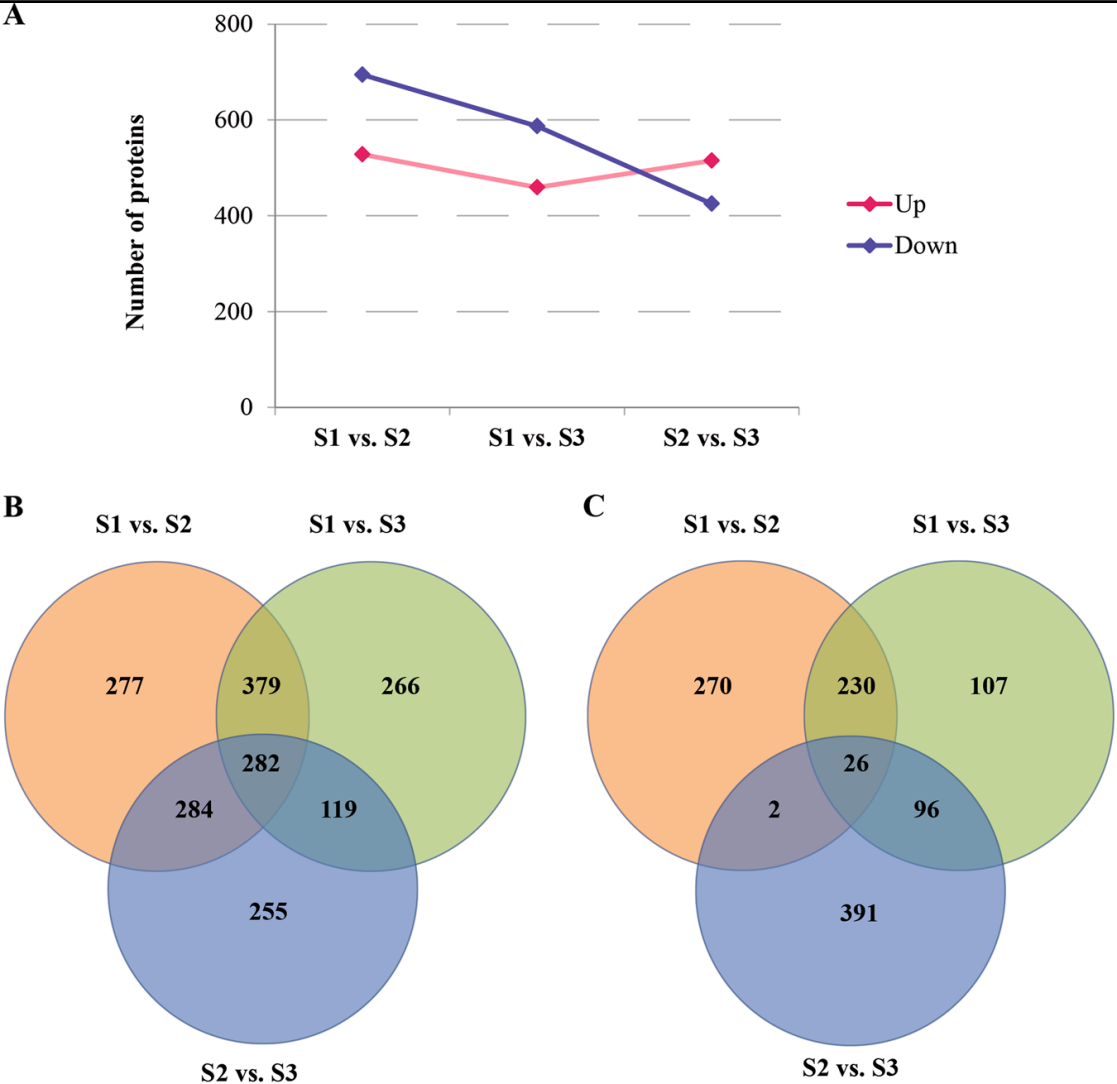
The characteristic of DEPs distribution in three libraries. **a** The number of DEPs in any two different stages. **b** Venn diagrams of DEPs mutual relationship among three libraries. **c** Venn diagrams of up-regulated proteins mutual relationship among three libraries

Base on the expression level of proteins, a FC value >1.2 or less than 0.83 with *P*-value < 0.05 were used as thresholds to judge DEPs. Using these standards, a total of 1222, 1046, and 940 DEPs were detected from S1 vs. S2, S1 vs. S3, and S2 vs. S3 pairs, respectively. Among these, totally 528, 459, and 515 proteins were up-regulated, and 694, 587, and 425 proteins were down-regulated in S1 vs. S2, S1 vs. S3, and S2 vs. S3 pairs, respectively (Fig. 2a, b; Supplementary Table S2). A total of 282 DEPs including 26 up-regulated proteins were presented among three comparison pairs (Fig. 2b), while 270, 107, and 391 DEPs were specifically expressed in up-regulated pairs of S1 vs. S2, S1 vs. S3, and S2 vs. S3, respectively (Fig. 2c). These results suggested that the specifically expressed proteins corresponding to S1 vs. S2, S1 vs. S3, and S2 vs. S3 pairs played certain roles on cortex splitting, primary expanding and secondary expanding in radish.

To further identify the DEPs involved in taproot thickening in radish, two comparison pairs were overlapped. For instance, we overlapped between S1 vs. S2 and S1 vs. S3 pairs for identifying proteins related to taproot thickening initiation, and overlapped between S1 vs. S2 and S2 vs. S3 pairs for identifying proteins related to taproot thickening process. More interestingly, totally 256 up-regulated proteins including 77 unknown proteins displayed overlapped accumulation in S1 vs. S2 and S1 vs. S3 pairs, whereas 122 up-regulated proteins including 43 unknown proteins displayed overlapped accumulation in S1 vs. S3 and S2 vs. S3 pairs (Supplementary Table S3). Our findings suggest that these proteins may play critical roles in taproot thickening initiation and taproot formation in radish.

### GO and pathway enrichment of DEPs

We used GO and pathway enrichment analysis of DEPs to determine significantly enriched DEPs compared with the background of all DEPs at *P*-value < 0.05 as the threshold value, and thereby explored the main biological functions, biochemistry metabolism, and signal transduction pathways.

In the S1 vs. S2 comparison, GO enrichment analysis showed that 1154, 1005, and 1090 DEPs were assigned to 271, 582, and 1708 GO terms of cell component, molecular function, and biology process, respectively. Among these, 83, 110, and 377 GO terms were significantly enriched in cell component, molecular function, and biology process, respectively (Supplementary Table S4). In the S1 vs. S3 comparison, 993, 864, and 944 DEPs were annotated to 254, 549, and 1689 GO terms of cell component, molecular function, and biology process, respectively. Of these, 76, 125, and 408 GO terms were significantly enriched in cell component, molecular function, and biology process, respectively (Supplementary Table S4). In the S2 vs. S3 comparison, 882, 742, and 833 DEPs were assigned to 250, 513, and 1601 GO terms of cell component, molecular function, and biology process, respectively. Of all, 65, 76, and 219 GO terms were significantly enriched in cell component, molecular function, and biology process, respectively (Supplementary Table S4). In addition, the shared significantly enriched GO terms of three libraries (Cluster frequency ≥50%) were calculated, and cytoplasm, cytoplasmic part, catalytic activity, metabolic process, cellular metabolic process, response to stimulus, and single-organism metabolic process were obtained.

Pathway enrichment analysis showed that 26, 24, and 14 pathways were significantly enriched in the S1 vs. S2, S1 vs. S3, and S2 vs. S3 comparison, respectively (Supplementary Table S5). The significantly enriched pathways of three libraries were “Metabolic pathways” (ko01100), “Glycolysis/Gluconeogenesis” (ko00010), “Biosynthesis of secondary metabolites” (ko01110), “Cysteine and methionine metabolism” (ko00270), “Glutathione metabolism” (ko00480), “Phenylalanine metabolism” (ko00360), and “Ribosome” (ko03010) (Table 1). In addition, several DEPs were identified to be involved in “Plant hormone signal transduction” (ko04075) and “Starch and sucrose metabolism” (ko00500) (Supplementary Table S6).

**Table 1:** The significant enrichment pathways for DEPs in three taproot thickening stages in radish

Terms	*P*-Value			Pathway ID
	S1 vs. S2	S1 vs. S3	S2 vs. S3	
**Specifically enrichment in S1 vs. S2 pair**				
Tyrosine metabolism	0.002338993	–	–	ko00350
Pyruvate metabolism	0.003318677	–	–	ko00640
Isoquinoline alkaloid biosynthesis	0.005839398	–	–	ko00950
Tropane, piperidine, and pyridine alkaloid biosynthesis	0.02102198	–	–	ko00960
Phenylalanine, tyrosine, and tryptophan biosynthesis	0.02465798	–	–	ko00400
Fatty acid metabolism	0.02649325	–	–	ko00071
beta-Alanine metabolism	0.03322849	–	–	ko00410
Phagosome	0.04160965	–	–	ko04145
**Specifically enrichment in S1 vs. S3 pair**				
Photosynthesis	–	2.23E−06	–	ko00195
Phenylpropanoid biosynthesis	–	0.001141693	–	ko00940
Arachidonic acid metabolism	–	0.00592627	–	ko00590
Linoleic acid metabolism	–	0.01070429	–	ko00591
Oxidative phosphorylation	–	0.01803258	–	ko00190
Citrate cycle (TCA cycle)	–	0.04408532	–	ko00020
**Specifically enrichment in S2 vs. S3 pair**				
Amino sugar and nucleotide sugar metabolism	–	–	0.007055244	ko00520
Glycosphingolipid biosynthesis	–	–	0.02370965	ko00603
Ether lipid metabolism	–	–	0.03840109	ko00565
**S1 vs. S2 and S1 vs. S3 pair share**				
Tryptophan metabolism	0.003174937	0.003847518	–	ko00380
Fructose and mannose metabolism	0.003546047	0.009507623	–	ko00051
Ubiquinone and other terpenoid–quinone biosynthesis	0.008722774	0.002321026	–	ko00130
Nitrogen metabolism	0.0111143	0.02068339	–	ko00910
Glyoxylate and dicarboxylate metabolism	0.01315227	0.00145352	–	ko00630
Pentose phosphate pathway	0.0236223	0.04274795	–	ko00030
Glucosinolate biosynthesis	0.03062338	0.03704522	–	ko00966
Carbon fixation in photosynthetic organisms	0.03135075	3.75E−05	–	ko00710
Arginine and proline metabolism	0.03617507	0.01826023	–	ko00330
**S1 vs. S2 and S2 vs. S3 pair share**				
Propanoate metabolism	0.003318677	–	0.0138437	ko00640
Alanine, aspartate, and glutamate metabolism	0.02117078	–	0.003866935	ko00250
**S1 vs. S3 and S2 vs. S3 pair share**				
Cyanoamino acid metabolism	–	1.69E−05	0.03116905	ko00460
alpha-Linolenic acid metabolism	–	0.000614791	0.009060137	ko00592
**S1 vs. S2, S1 vs. S3, and S2 vs. S3 share**				
Metabolic pathways	6.47E−06	6.29E−14	0.000324501	ko01100
Glycolysis/gluconeogenesis	6.56E−06	0.000968121	0.006929212	ko00010
Biosynthesis of secondary metabolites	9.98E−06	3.34E−06	0.001973937	ko01110
Cysteine and methionine metabolism	0.000191997	0.002653402	0.000150698	ko00270
Ribosome	0.007452515	0.03416924	0.03002672	ko03010
Glutathione metabolism	0.008876445	0.0001046	0.003305723	ko00480
Phenylalanine metabolism	0.03617507	0.004421169	0.03331485	ko00360

### Associated analysis of mRNA and proteins during taproot thickening in radish

**Fig. 3:**
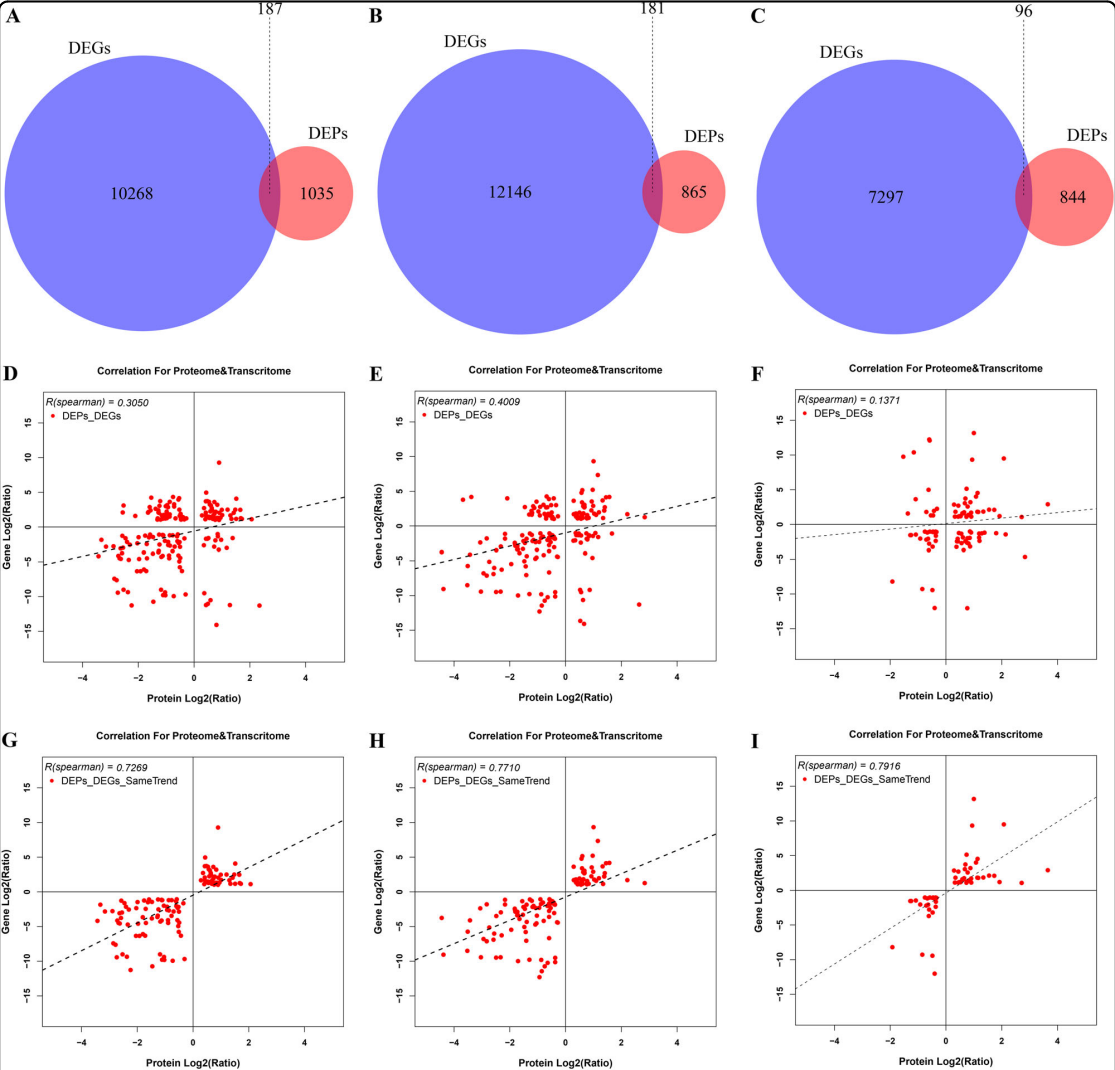
Association results of proteome and transcriptome in three libraries. **a**–**c** Venn diagram of associated DEPs/DEGs. **d**–**f** *R* value of DEPs and DEGs association. **g**–**i** *R* value of the same trends DEPs/DEGs. **a**, **d**, **g** S1 vs. S2 pair. **b**, **e**, **h** S1 vs. S3 pair. **c**, **f**, **i** S2 vs. S3 pair. *R* is the Pearson correlation coefficient

**Fig. 4:**
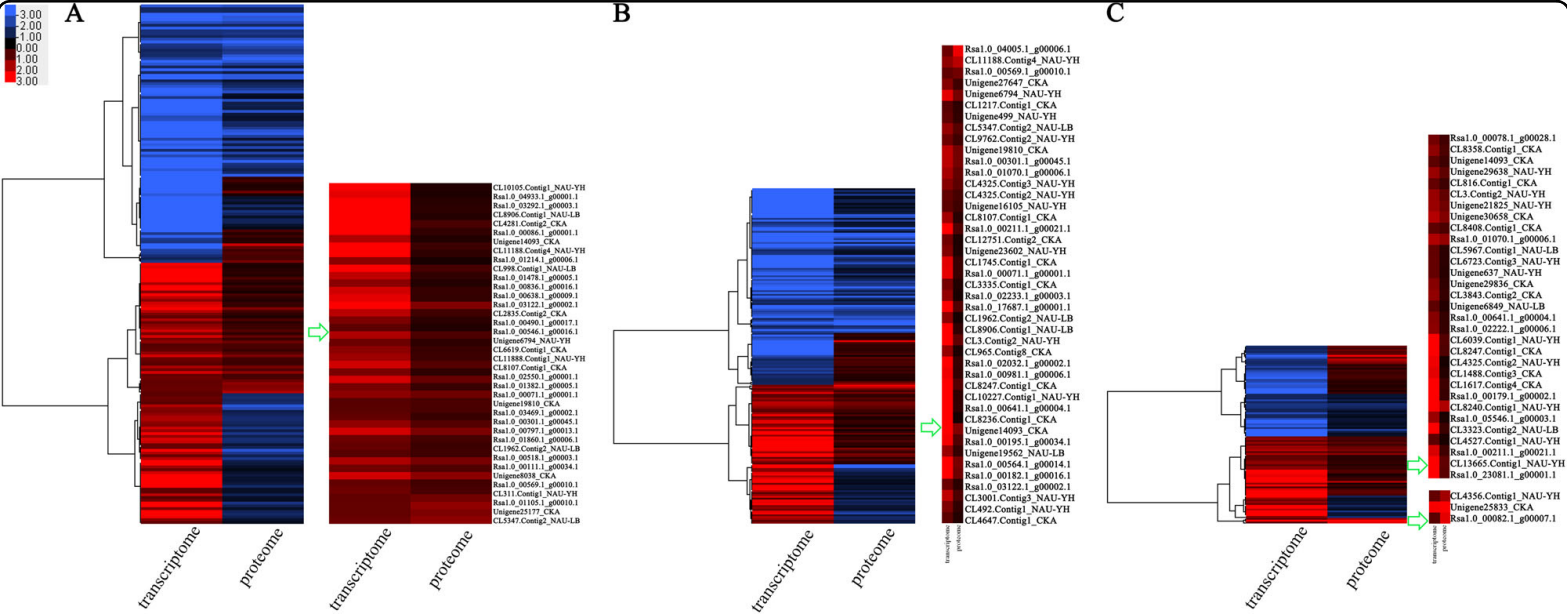
Cluster diagram for the concordance between proteome and transcriptome based on pairwise comparison among the three libraries. **a** S1 vs. S2 pair. **b** S1 vs. S3 pair. **c** S2 vs. S3 pair

Proteome and transcriptome analysis showed two difference levels, which reflect genes expression. In order to mutual corroborate the data reliability, association analysis of DEPs data with previous transcriptome data was performed in this study (Supplementary Table S7)^14^. The result showed that 187, 181, and 96 DEPs were successfully matched with DEGs in the pairs of S1 vs. S2, S1 vs. S3, and S2 vs. S3, respectively (Fig. 3a–c; Supplementary Table S8), and the corresponding Spearman correlation coefficient for proteome and transcriptome (*R, Spearman*) were 0.3050, 0.4009, and 0.1371, respectively (Fig. 3d–f). These DEPs/DEGs results could be categorized into four groups: (i) the expression patterns of DEPs and DEGs with both up-regulated (up-DEPs & up-DEGs; 47, S1 vs. S2; 43, S1 vs. S3; 35, S2 vs. S3); (ii) the expression patterns of DEPs and DEGs with both down-regulated (down-DEPs & down-DEGs; 74, S1 vs. S2; 78, S1 vs. S3; 23, S2 vs. S3); (iii) the expression patterns of DEPs and DEGs with the opposite, i.e., either up-regulated DEPs and down-regulated DEGs (up-DEPs & down-DEGs; 19, S1 vs. S2; 28, S1 vs. S3; 26, S2 vs. S3), or down-regulated DEPs and up-regulated DEGs (down-DEPs & up-DEGs; 47, S1 vs. S2; 32, S1 vs. S3; 12, S2 vs. S3). Interestingly, although the Spearman correlation coefficient between DEPs and DEGs was weak (0.3050, S1 vs. S2; 0.4009, S1 vs. S3; 0.1371, S2 vs. S3), the same expression trends of DEPs and DEGs were relatively strong among the association results (0.7269, S1 vs. S2; 0.7710, S1 vs. S3; 0.7916, S2 vs. S3) (Fig. 3g–i). The result may suggest that gene regulation mechanisms were different in mRNA and protein level, and the associated proteins/genes were critical for exploring the molecular mechanism of taproot thickening in radish. Furthermore, the cluster analysis was performed to identify the characteristic of correlation between transcriptome and proteome (Fig. 4).

### RT-qPCR validation

**Fig. 5:**
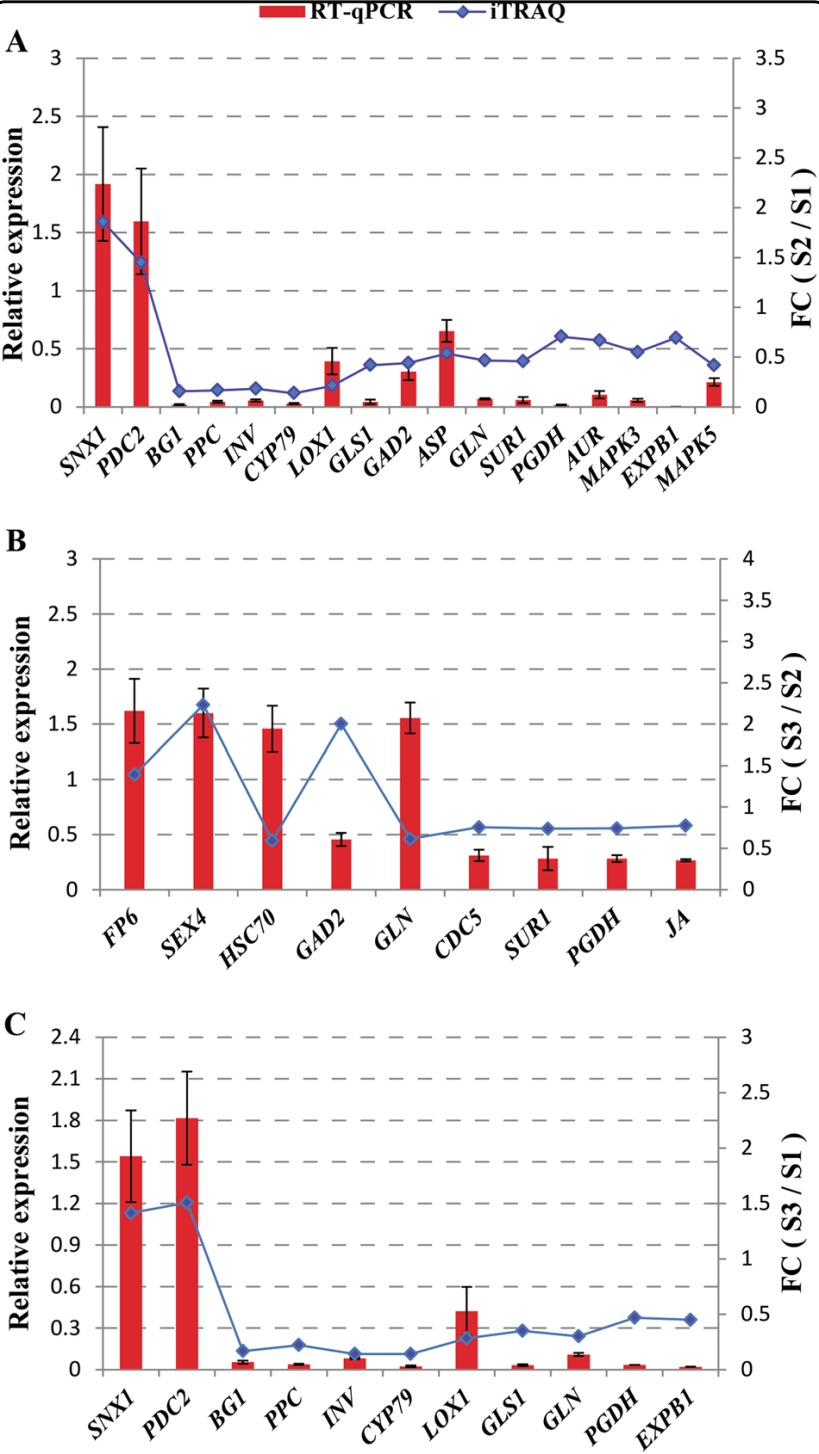
Validation of the mRNA expression levels of proteins from iTRAQ results by RT-qPCR. **a** S1 vs. S2 pair. **b** S1 vs. S3 pair. **c** S2 vs. S3 pair

To evaluate the validity of iTRAQ results, 17, 11, and 9 proteins from three pairwise comparisons (S1 vs. S2, S1 vs. S3, and S2 vs. S3) were randomly selected and detected with RT-qPCR analysis. The related transcript of these proteins include signaling transduction (MAPK3, MAPK5, SUR1, AUR, JA, SNX1, LOX1), cell division and expanding (CDC5, EXPB1), sucrose biosynthesis and metabolism (PDC2, BG1, PPC, INV, SEX4, PGDH), amino acid biosynthesis and metabolism (GLN, ASP, GAD2, GLS1, ASP), HSP (HSC70), FP6 and CYP79F1 (Supplementary Table S1). As shown in Fig. 5, the transcript levels of BG1, PPC, INV, CYP79F1, LOX1, GLS1, GLN, PGDH, and EXPB1 were higher in the pre-cortex splitting stage than in the cortex splitting stage and expanding stage, whereas SNX1 and PDC2 were higher in the cortex splitting stage and expanding stage than in the pre-cortex splitting stage at mRNA levels. Interestingly, the transcript levels of FP6, SEX4, HSC70, and GLN were higher in the expanding stage than in the pre-cortex splitting stage and cortex splitting stage, while the transcript of CDC5 was higher in cortex splitting stage than in the expanding stage. Furthermore, the mRNA levels of signaling transduction proteins including MAPK3, MAPK5, and AUR were higher in the pre-cortex splitting stage than in the cortex splitting stage. Overall, it was found that there was a good concordance between iTRAQ and mRNA expression levels (Fig. 5), indicating the reliability of iTRAQ-based quantitative proteomic data analysis.

## Discussion

**Fig. 6:**
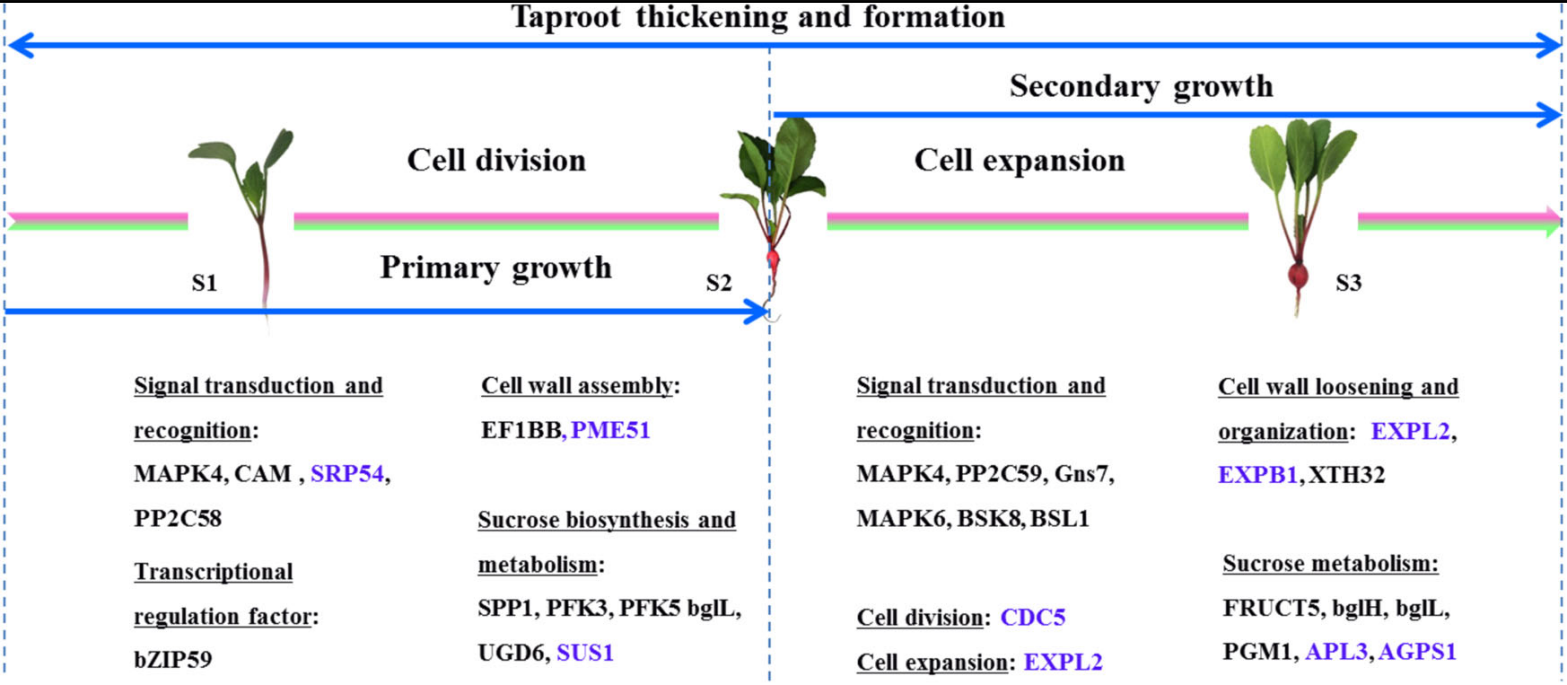
An overview of proteome changes involved in radish taproot thickening at three developmental stages. The blue font represents overlap proteins between DEPs and DEGs, and the normal font represents unique proteins in this study. MAPK4 mitogen-activated protein kinase 4, CAM putative calmodulin, SRP54 signal recognition particle subunit, PP2C58 probable protein phosphatase 2C 58, SPP1 sucrose-phosphatase 1, PFK3 6-phosphofructokinase 3, PFK5 6-phosphofructokinase 5, bglL beta-glucosidase L, UGD1 UDP-glucose 1-dehydrogenase, SUS1 sucrose synthase 1, PME51 phosphoglucomutase 1, bZIP59 bZIP transcription factor 59, EF1BB elongation factor 1B alpha-subunit 2, PP2C59 protein phosphatase 2C 59, Gns7 glucan endo-1,3-beta-glucosidase 7, MAPK6 mitogen-activated protein kinase 6, BSK8 brassionsteroid-signaling kinase 8, BSL1 serine/threonine-protein phosphatase, EXPL2 expansin-like A2, EXPB1 expansion B1, XTH24/XTH32 xyloglucan endotransglucosylase 24/32, FRUCT5 beta-fructofuranosidase, bglH beta-glucosidase H, PGM1 phosphoglucomutase 1, APL3 glucose-1-phosphate adenylyltransferase, bglI beta-glucosidase I, CDC5 cell division cycle 5-like protein

As an important storage organ, radish taproot thickening determines its final yield and quality of the product. miRNA and mRNA of radish taproot thickening at pre-cortex splitting stage (S1), cortex splitting stage (S2), and expanding stage (S3) have been studied, which provided novel insights into the genetic regulatory network during taproot thickening^9,14^. However, the molecular mechanism underlying taproot thickening in radish is far from being fully clarified. In this study, iTRAQ-based proteomic approach was employed to monitor DEPs from three different stages of radish taproot thickening (S1, S2, and S3). Furthermore, based on the integrative analysis of transcriptomic and proteomic data, an overview of proteome changes involved in radish taproot thickening at three developmental stages was put forward (Fig. 6). To the best of our knowledge, this is the first comprehensive investigation to characterize the potential functional proteins involved in taproot thickening in radish.

### Proteomic studies could be a supplement to transcriptome analysis

In this study, iTRAQ-based proteomic approach was employed to identify DEPs from three different stages of radish taproot thickening (S1, S2, and S3), as a follow-up to previous study^9,14^, which could be beneficial to further dissecting the genetic molecular mechanism underlying radish taproot thickening. Furthermore, for further screening critical genes involved in taproot formation in radish, association analysis between DEGs and DEPs in three development stages of taproot thickening was also performed in this study. The relatively weak correlation of genes at mRNA and protein level indicates that an alteration in a proportion of transcripts may not be translated into changes in protein abundance. Low congruency of genes in mRNA and protein level was similar with other previous studies^20,35^.

As shown in Fig. 6, the blue font indicated the proteins with the same expression patterns in mRNA and protein level, whereas the normal font represented unique proteins. For example, proteins involved in cell wall loosening and reconstruction including XTH24 and XTH32 and proteins involved in signal transduction such as PP2C58, BSK8, and BSL1 were unique proteins that were only identified in this study but not gathered in transcriptome analysis. The results showed that the transcriptome and proteome levels were various under taproot thickening in radish, and it could be presumed that proteomic studies was used as an effective supplement to transcriptome analysis.

### Phytohormone is a basic regulatory factor for taproot thickening

Phytohormone is critical for the growth modulation of the root system^36^. Five main hormones including auxin, cytokinin, ethylene, abscisic acid (ABA), and gibberellic acid (GA) were extensively investigated in plant root system. Auxin-to-cytokinins ratio regulates root and shoot meristem. In general, the high ratio is favorable for the root meristem and control of root architecture, while the lower ratio is conducive to shoot meristem. The auxin influx carriers including AUX1, PGP, LAX, and auxin efflux transporters such as ABC transporters affect the distribution of auxin and regulate root morphogenesis^37^. Previous studies showed that changing the level of auxin influences cytokinin concentration, which functioned by the mechanism of relevant proteins controlling hormone accumulation^38^. Several other hormones including ethylene, ABA, and GA are also responsible for root development. For example, ethylene inhibits adventitious root formation in Arabidopsis^39^, ABA could modulate main root elongation in response to drought, and GA could enhance cell elongation of root. In radish plants, cytokinin plays critical role in controlling developmental process of taproot thickening^7^. In this study, “plant hormone signal transduction” is one of the most enriched pathways, and several DEPs including Gns7 (glucan endo-1,3-beta-glucosidase 7, Rsa1.0_00048.1_g00023.1), AHP2 (histidine-containing phosphotransfer protein 2, Rsa1.0_00568.1_g00001.1), PYR1 (abscisic acid receptor, CL14734.Contig1_CKA, CL3244.Contig2_NAU-LB), and PP2C58 (protein phosphatase 2C 58, CL6992.Contig1_NAU-LB) were up-regulated in this process, suggesting that these proteins would be likely involved in the regulation of radish taproot thickening (Supplementary Table S6). However, a system network of hormone interactions in radish taproot thickening needs to be further investigated.

### Sucrose metabolism provides substance basis for taproot thickening

Sucrose is not only a product of plants photosynthesis, but also a carbon source involved in other important metabolite compounds synthesis such as starch, cellulose and proteins. In this study, several candidate proteins including cell wall-related proteins (e.g., CSLD2, PME17, PME18, PME34, and PME51) and energy metabolism proteins (e.g., BXL, FRK, PGM, TPS7, PHS, BXL, CSLD2, MA1, SUS1, BFRUCT, BAM, APL, FRUCT5, BGLU, UGD1, HXK1, PGIC, and AGPS1) were identified to be differently expressed in three libraries (Supplementary Table S6). Interestingly, PME51 (pectinesterase/pectinesterase inhibitor 51, Rsa1.0_01105.1_g00010.1) and FRK5 (fructokinase 5, CL4819.Contig2_CKA) were up-regulated in S1 vs. S2 pair, FRK1 (fructokinase 1, Rsa1.0_03943.1_g00005.1) was up-regulated both in S1 vs. S2 and S1 vs. S3 pairs, and PHS2 (alpha-glucan phosphorylase 2, CL13665.Contig1_NAU-YH), BAM3 (Beta- amylase 3, Rsa1.0_00524.1_g00011.1), APL2 (Glucose-1-phosphate adenylyltransferase, Rsa1.0_00583.1_g00001.1) and FRUCT5 (Beta-fructofuranosidase, Unigene25061_NAU-YH) were up-regulated in S2 vs. S3 pair, suggesting that these DEPs might be involved in cortex splitting, primary growth, and secondary growth of radish taproot thickening, respectively (Supplementary Table S6). In the present study, sucrose synthase 1 (SUS1) was down-regulated in S3 vs. S1 comparison pair, which had the same trend with transcriptomic data of radish with small size taproot (radish type: cherry radish)^14^ but exhibited a conflict trend with result from long and thick taproot radish^11^. It was inferred that the different expression patterns of SUS1 gene between large- and small-sized radish might lead to sucrose unloading capacity difference, which partially contribute to the generation of different taproot sizes and production.

Interestingly, the basic formation mechanism of the root and tuber crops (RTCs) could be interlinked, but the genes and proteins involved in these processes may be diverse. For example, sporamin including sporamin A and sporamin B were specifically identified to be up-regulated from storage roots in sweet potato^17^, and patatin proteins (patatin protein 13 and patatin protein 15) were specifically identified from potato^16^. The sporamin and patatin protein were not identified from taproot thickening in radish, whereas several proteins including SUS1, EXPL2, XTH32, and CDC5 were specifically identified to be significantly differentially expressed in radish, suggesting that there is certain specificity in different types of RTCs (Fig. 6; Supplementary Table S2). However, carbohydrate metabolism was one of the most important pathways in the development of RTCs, and the proteins involved in this process including fructokinase, phosphoglycerate kinase, NADH dehydrogenase, glyceraldehyde-3-phosphate dehydrogenase, fructose-bisphosphate aldolase, ADP-glucose pyrophosphorylase, glucose-1-phosphate adenylyltransferase, and alcohol dehydrogenase were identified to be differentially expressed both in potato^16^ and radish (Supplementary Table S2). Meanwhile, several proteins including protein disulfide isomerase and anionic peroxidase were identified to be significantly expressed both in storage root of sweet potato^17^ and radish taproot thickening (Supplementary Table S2), indicating that the protein expression level in the energy supply was relatively conservative. Therefore, we speculated that the possible reason is the difference in proteins to be involved resulting in diverse accumulation of substances in potato, sweet potato, and radish.

### Cell division and cell expansion determining taproot thickening

Taproot thickening is a process characterized by changes in substances and energy. During this period, the basic metabolism of the taproot was reduced, and it would be act as a storage bank. A series of proteins are involved in the biosynthesis and metabolism of sugar, starch, and protein, which play critical roles in the initial step of taproot thickening in radish^13,40,41^. In the present study, cell division cycle 5-like protein (CDC5), a DNA binding protein belonging to a member of MYB3R- and R2R3-type, was up-regulated in S1 vs. S2 pair, consistent with its action in *Arabidopsis*^42–45^. Interestingly, *CDC5* gene was also identified to be up-regulated in S1 vs. S2 pair^14^. Meanwhile, cell division and cell enlargement tend to be along with the cell wall loosening and reconstruction, and expansin (EXP) and xyloglucan endotransglucosylase/hydrolase (XTH) proteins played critical roles in these processes. Previous studies showed that down-regulation of *EXPB1* gene enhanced storage root development in sweet potato^46^, and *XTH24* gene involved in cell wall elongation in Arabidopsis^47^. In this study, the expansin proteins including EXPB1 (expansin B1) and EXLA2 (expansin-like A2) were identified to be differentially expressed, among them EXLA2 was all up-regulated in S1 vs. S2 pair and S1 vs. S3 pair, whereas EXPB1 was down-regulated in S1 vs. S2, all in line with gene expression pattern at mRNA level (Fig. 6; Table 2)^14^. Moreover, XTH32 was up-regulated in S2 vs. S3 pair, while XTH24 that involved in GA-mediated signaling pathway, cell wall loosening, organization or biogenesis, and xyloglucan metabolic process was down-regulated in S1 vs. S2 pair and S1 vs. S3 pair (Fig. 6; Table 2). The results suggested that CDC5, EXPB1, and XTH24 might play critical roles in taproot thickening process in radish.

**Table 2:** Identification of critical DEPs involved in taproot thickening in radish

In addition, previous studies illustrated that transcription factors (TFs) including MADS-box, ABF/AREB, and homeobox were also responsible for the formation of modified stems or storage roots^38^. In the present study, MYB and bZIP TFs were both up-regulated in S1 vs. S2 pair, suggesting that MYB and bZIP might play critical roles in regulating the development of taproot thickening in radish, which were consistent with their action of rhizome formation in lotus root^14,42^.

In conclusion, the comparative proteome changes among three developmental stages of taproot thickening were systematically investigated in radish. A total of 1862 DEPs were identified during radish taproot thickening. GO and pathway enrichment analysis showed that several DEPs were mainly involved in “plant hormone signal transduction”, “starch and sucrose metabolism” and “biosynthesis of secondary metabolites”. Furthermore, the integrative analysis of DEGs and DEPs data enhanced our understanding of taproot thickening molecular mechanisms in radish. Overall, it was concluded that taproot thickening initiation was triggered by phytohormone, and several functional proteins including CDC5, EXPB1, and XTH24 could contribute to cell division and expansion during radish taproot thickening. Together, these findings would provide fundamental insights into comprehensive clarification of molecular regulatory network of taproot thickening in radish.

## Electronic supplementary material

Table S1

Table S2

Table S3

Table S4

Table S5

Table S6

Table S7

Table S8

Figure S1

Figure S2
