# Supplementary material for: Comparative proteomic analysis provides insight into a complex regulatory network of taproot formation in radish (Raphanus sativus L.)
Source: Hortic Res. 2018 Oct 1;5:51. doi: 10.1038/s41438-018-0057-7 (PMC6165848; doi:10.1038/s41438-018-0057-7)
Supplement: Supplementary file 9 — Figure S1 [file 41438_2018_57_MOESM9_ESM.docx]

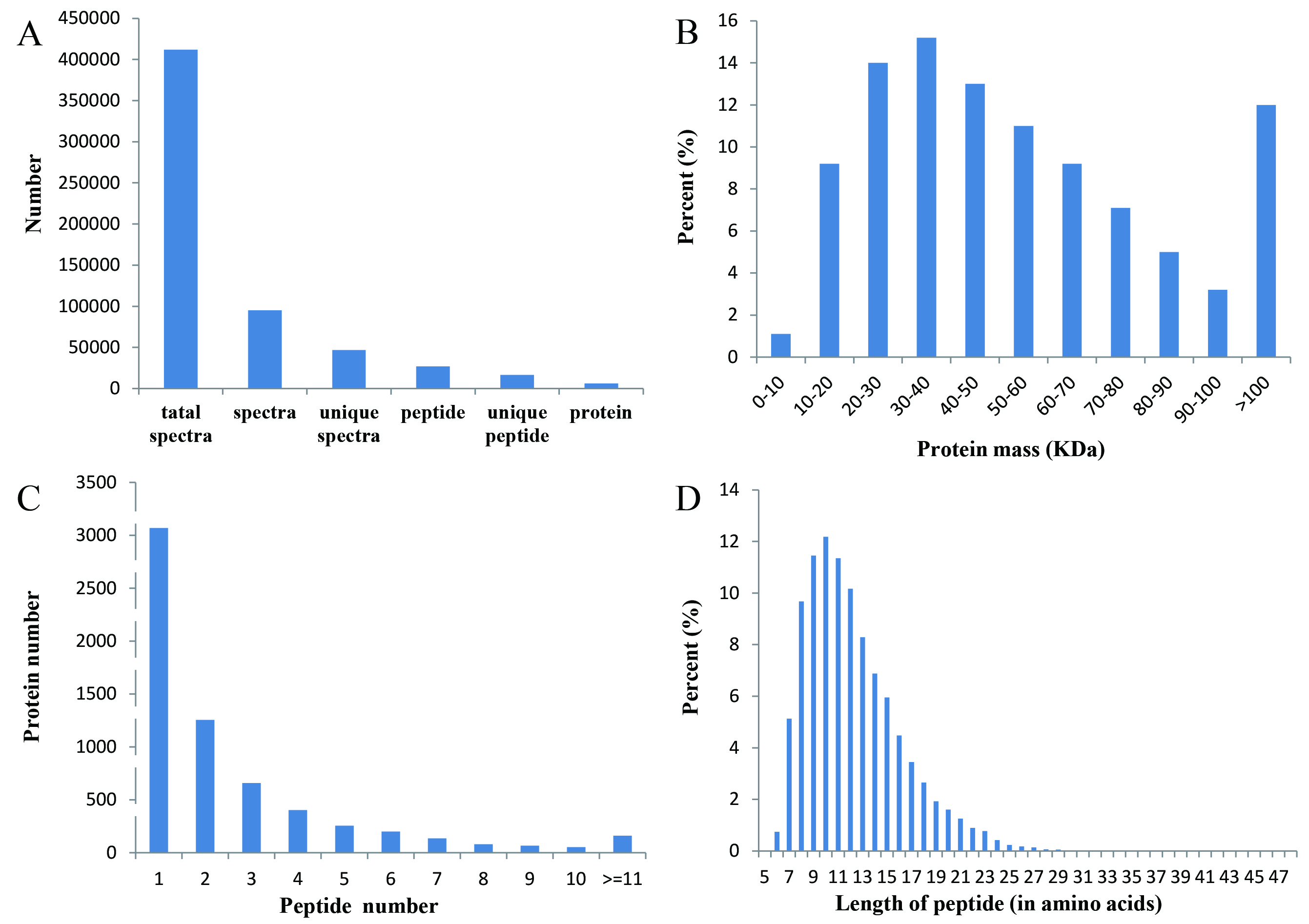


Figure S1 The basic information statistics of the identified proteins. a Proteome identification. b The distribution of protein mass. c The distribution of peptide number. d The distribution diagram of peptide length
