# Supplementary figures and images for: Comparative proteomic analysis provides insight into a complex regulatory network of taproot formation in radish (Raphanus sativus L.)
Source: Hortic Res. 2018 Oct 1;5:51. doi: 10.1038/s41438-018-0057-7 (PMC6165848; doi:10.1038/s41438-018-0057-7)

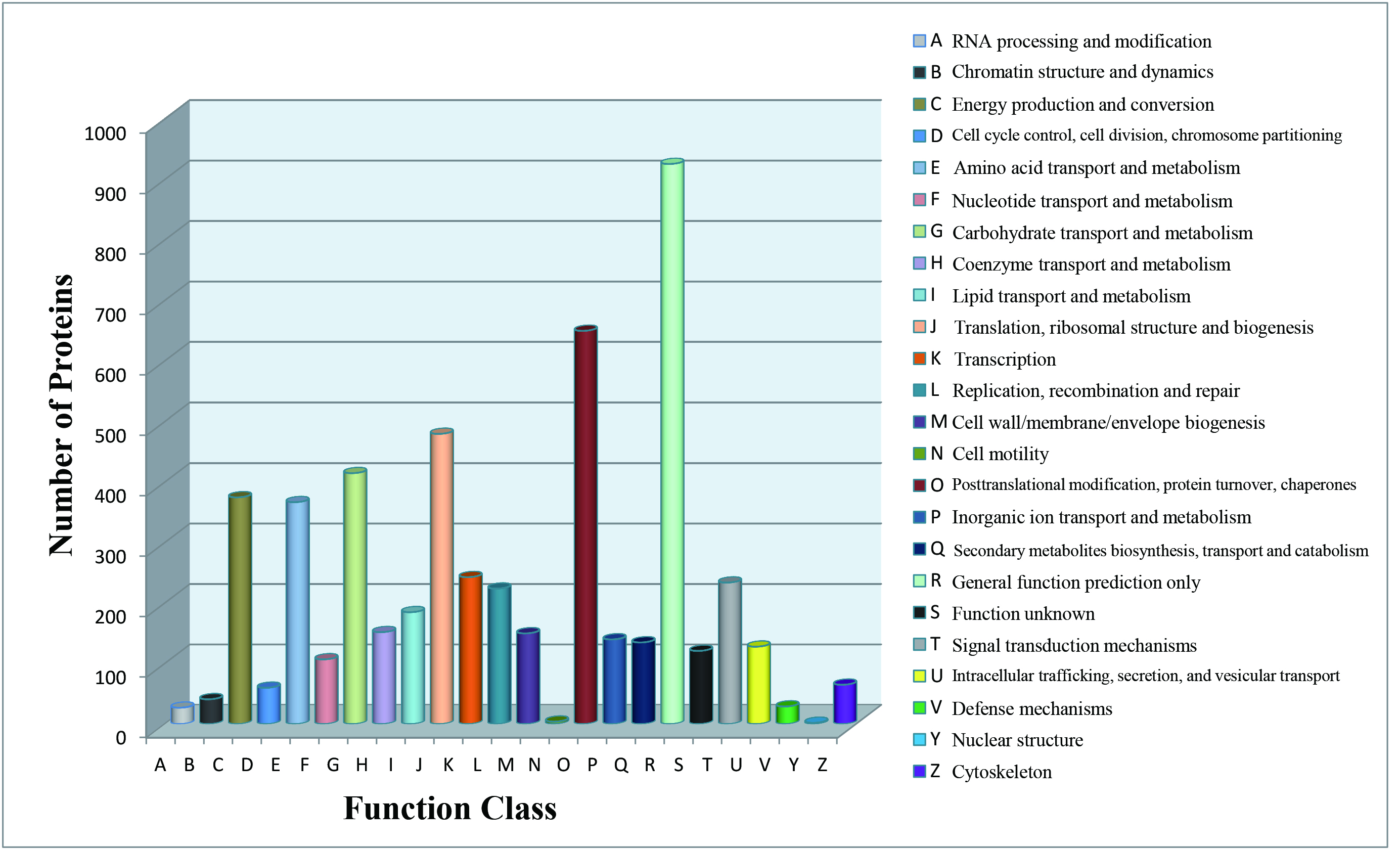


Figure S2 COG functional classification for the identified proteins

Supplement: Supplementary file 10 — Figure S2 [file 41438_2018_57_MOESM10_ESM.docx]
